# Supplementary figures and images for: Prioritization of neglected tropical zoonotic diseases: A one health perspective from Tigray region, Northern Ethiopia
Source: PLoS One. 2021 Jul 22;16(7):e0254071. doi: 10.1371/journal.pone.0254071 (PMC8297755; doi:10.1371/journal.pone.0254071)

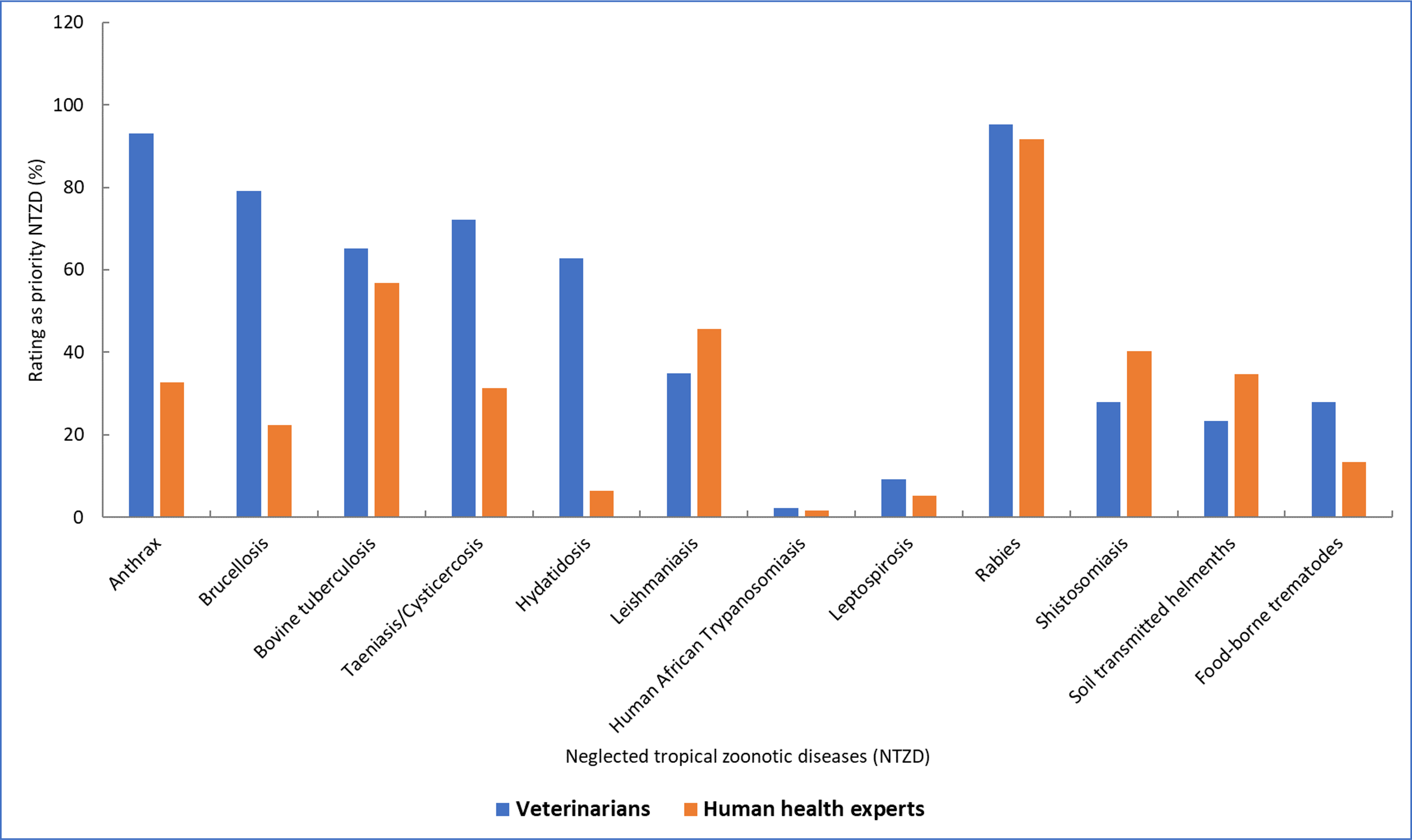

Supplement: S1 Fig — (TIF) [file pone.0254071.s001.tif]

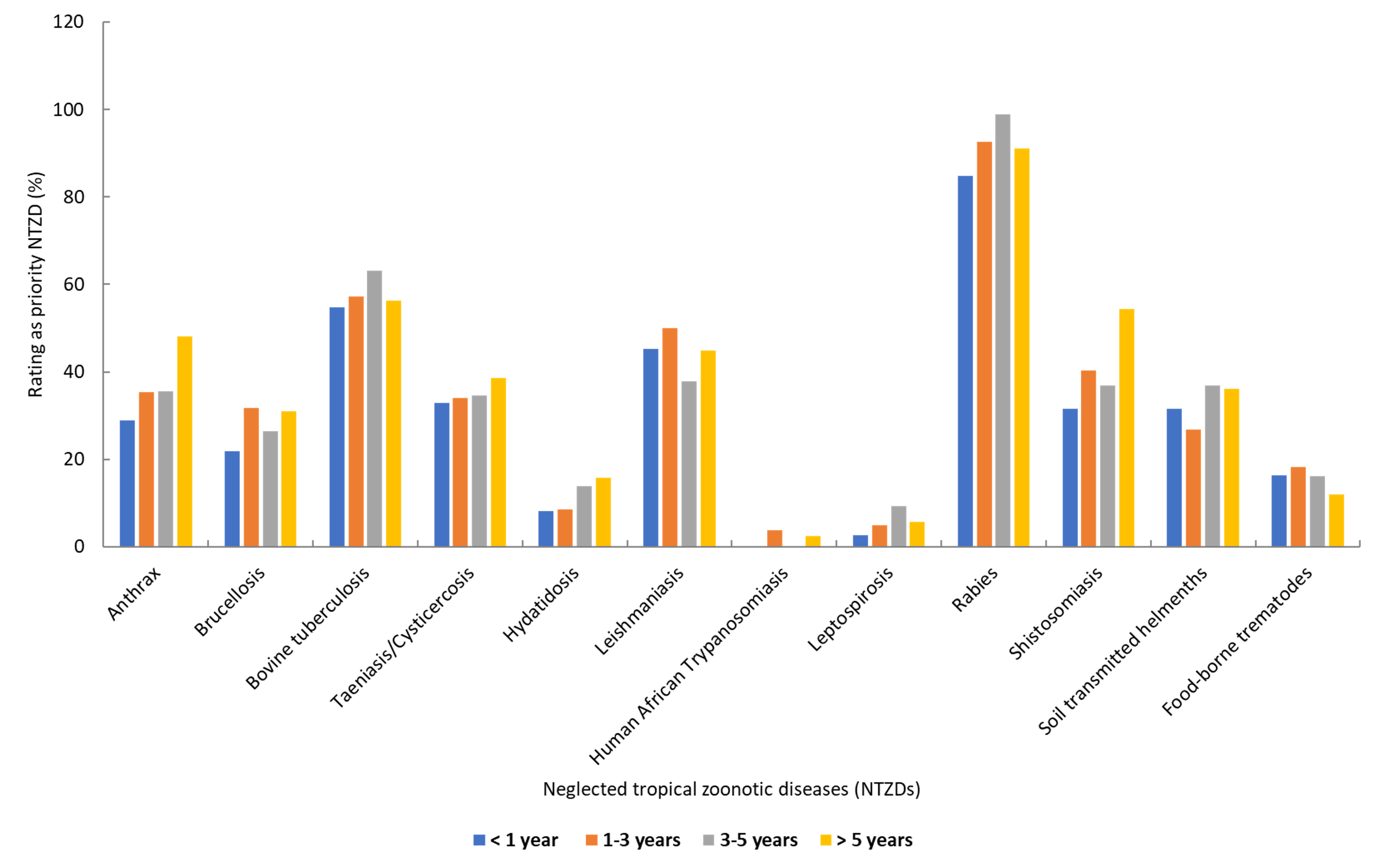

Supplement: S2 Fig — (TIF) [file pone.0254071.s002.tif]
