# Supplementary material for: Prioritization of neglected tropical zoonotic diseases: A one health perspective from Tigray region, Northern Ethiopia
Source: PLoS One. 2021 Jul 22;16(7):e0254071. doi: 10.1371/journal.pone.0254071 (PMC8297755; doi:10.1371/journal.pone.0254071)
Supplement: S1 File — (PDF) [file pone.0254071.s003.pdf]

Questionnaire for professionals

Part I: Demographic Characteristics of the respondents

1. Age (yrs):-----------------------
2. Sex: o. Male 1. Female
3. Address :-Zone ---------------------------------
4. Organization ---------------------------------
5. Job Title ------------------------------------
6. How long have you been in your job? 0<1 year 1. 1 to 3 years 2. 3to 5 years 3. >5 years

Part II: Specific research based questions

7. What are the major NTZD in your zone? (Multiple answer is possible)

0Anthrax	5. Leishmaniasis	10. Soil transmitted helminthes
1. Brucellosis	6.Human Africa Trypanosomiasis	11 Food born trematods
2. Bovine tuberculosis	7 Leptospirosis	
3. T. Saginata cysticercosis	8 Rabies	
8 Cysticechnococosis (hydatidiosis)	9 Schistosomiasis	

8. What are the major NTZD in your zone (in your work place)?

List of NTZD	Rank burden wise				
Anthrax	V. High	High	Moderate	Low	V. Low
Brucellosis					
Bovine tuberculosis					
T.saginata (Cysticercosis)					
Cystic echnococosis (hydatidiosis)					
Leishmaniasis					

Human African trypanosomiasis					
Leptospirosis					
Rabies					
Schistosomiasis					
Soil transmitted Helminthes					

9. What do you about the major causes (risk factors)that may aggravate the distribution and burden of NTZD ? (Multiple answer is possible)

List of NTZD	Major risk factors					
	Consuming raw animal products	Contact with animal, animal by-product/waste	Favorable environment	Sharing the same environment with animal	Contact with contaminated water	Lack of awareness
Anthrax						
Brucellosis						
Bovine tuberculosis						
T.saginata (Cysticercosis)						
Cystic echnococosis (hydatidiosis)						
Leishmaniasis						
Human African trypanosomiasis						
Leptospirosis						
Rabies						
Schistosomiasis						
Soil transmitted Helminthes						
Food born trematods						

10. What do you think about the mode of transmission?

List of NTZD	Major risk factors					
	Body fluid/blood contact	Direct contact (skin penetration)	Ingestion	Animal bite	Vectors and reservoirs	Others
Anthrax						
Brucellosis						
Bovine tuberculosis						
T.saginata (Cysticercosis)						
Cystic echnococosis (hydatidiosis)						
Leishmaniasis						
Human African trypanosomiasis						
Leptospirosis						
Rabies						
Schistosomiasis						
Soil transmitted Helminthes						
Food born trematods						

11. Did you get any opportunity to attended training on NTZD? 0. Yes 1. No
12. If yes, to Q11, when and on which topics?____________________________________

____

13. Do you provide awareness creation training about NTZD in your zone?

0 Yes 1. NO

14. If yes, to Q13, on which topics disease did you proved training?

____
