# Supplementary material for: Prioritization of neglected tropical zoonotic diseases: A one health perspective from Tigray region, Northern Ethiopia
Source: PLoS One. 2021 Jul 22;16(7):e0254071. doi: 10.1371/journal.pone.0254071 (PMC8297755; doi:10.1371/journal.pone.0254071)
Supplement: S2 File — (PDF) [file pone.0254071.s004.pdf]

FGD Discussion Points for Health Professionals (Human health and Veterinarians)

1. What is the impact of Neglected tropical zoonotic disease on socioeconomic and public health importance?
2. What is the distribution and burden of Neglected tropical zoonotic disease in Tigray region in general and in your zone in particular?
3. What are the major Neglected tropical zoonotic diseases in your zone list the top five?
4. What is the status of laboratory infrastructure, diagnostic technique and treatment efficacy in your zone?
5. What is the disease reporting system in your zone? And what seems like the institutional collaboration between human and animal health experts in your zone
6. What are the major risk factors of Neglected tropical zoonotic disease?
