# Supplementary material for: Prioritization of neglected tropical zoonotic diseases: A one health perspective from Tigray region, Northern Ethiopia
Source: PLoS One. 2021 Jul 22;16(7):e0254071. doi: 10.1371/journal.pone.0254071 (PMC8297755; doi:10.1371/journal.pone.0254071)
Supplement: S3 File — (PDF) [file pone.0254071.s005.pdf]

Tool for Prioritization of NTZD in Tigray Region, Northern Ethiopia

Inclusion Criteria of informants (Knowledge assessment criteria)

- ✓ Should have least one year work experience at the area with his profession
- ✓ Answered at least four of the questions listed below

1. Do you know about NYZD ? 0= N , 1 = Yes , 2+ have no idea
2. Do you know about NTZD major problem of your area 0=No, 1=Yes ,2=have no idea
3. (if yes) can you mention at least three of them ?____________________________
4. What are the mode of transmission to human

    0=Contact

    1= Ingestion (law milk , raw meat , egg, etc…..)

    2= inhalation

    3= No idea

5. Do you know animals play vital role for transmission of the diseases? 0=No, 1=Yes,

List Of NTZD	Severity of illness to humans score=1)			Transmission potential humans& animals (Score=0.65)			Economics burden of the disease (Score =0.65)							Inter- sect oral Collaboration (Score =0.45)			Availability of intervention(Scopre=0.30)			
	Case fatality rate humans without treatment? (Score=1)			Proportion of human disease attributable to animal exposure?(score =.85)			Case fatality rate in animals without treatment?(Score =0.23)			OIE rep. D+(s=0.22)		ELMP D+(s=0.20)								
	0-1% (s=0.33)	1-5% (s=0.67)	>5%(s=1)	H to H Continuous (s=0)	H to H w/t continuous	Continuous A to H (no H-	o-1%(s=0.33)	1-5%(s=0.67)	>5%(s=1)	Yes(s=1)	No(s=o)	Yes(s=1)	No(s=o)	Yes,	Yes , weak	No(s=0)	Ay . of animal	Ay . of animal	S+1Vaccine	Neither()
Anthrax																				
Brucellosis																				
Bovine tuberculosis																				
T.saginata (Cysticercosis)																				
Cystic echnococosis (hydatidiosis)																				
Leishmaniasis																				
Human African																				

trypanosomiasis																				
Leptospirosis																				
Rabies																				
Schistosomiasis																				
Soil transmitted Helminthes																				
Food born trematods																				
Rift valley fever																				
