# Supplementary material for: Prioritization of neglected tropical zoonotic diseases: A one health perspective from Tigray region, Northern Ethiopia
Source: PLoS One. 2021 Jul 22;16(7):e0254071. doi: 10.1371/journal.pone.0254071 (PMC8297755; doi:10.1371/journal.pone.0254071)
Supplement: Attachment — Submitted filename: Response to reviewers and editor_B.M.docx [file pone.0254071.s009.docx]

Date: 5^th^ May 2021

**Ref:** PONE-D-20-30909

**Manuscript Title:** “Prioritization of Neglected Tropical Zoonotic Diseases: A One Health Perspective from Tigray Region, Northern Ethiopia”.

Subject: **Submission of Revised manuscript (R1)**

Dear esteemed editorial team (PLOS ONE Journal),

Many thanks for considering our manuscript for publication after revision. We are very much thankful to the critical review by the esteemed reviewers and academic editor. All the comments forwarded by the esteemed reviewers and academic editor are critically reviewed and improvement has been made in our revised manuscript. We provide a point-by-point discussion below and have updated the manuscript with the track changes.

We would like also to thank the academic editor for considering our current situation (internet lockdown due to war) and extending the deadline for submitting our revised manuscript

I. **Response to Academic editor**

- Line 97 to 103, the subheading "Study area and population" was changed to “Study area description” (line 102 current version) and detailed description of the study area was given. The sampling techniques and reference population description were moved to the "Study design, Study Population and Sample size determination" subheading.
- Line 119 previous versions and line 141 current versions, representation for “Z” have been modified as per the editor comments.
- Description regarding the multivariate regression analysis technique was given focusing on model fitting, model building and quality parameters. The interpretation and presentation of odds ratio results is made according to standards and previous literature.
- The abbreviations used for zones were described in all tables and elsewhere
- Table on page 16 previous versions and page 18 on current version was edited as Table 4.

II. **Response to Journal Requirements:**
1. Please ensure that your manuscript meets PLOS ONE's style requirements, including those for file naming:

**Response:** The manuscript is prepared according to the ‘Manuscript Body Formatting Guidelines’ of PLOSE ONE

2. Survey or questionnaire related issue:

**Response:** Additional information on the questionnaire survey was included especially focusing on the pre-testing of the questionnaire. Moreover, the questionnaire used in this study is also attached as a supporting file (Line 167, current manuscript)

3. Please amend either the abstract on the online submission form or the abstract in the manuscript so that they are identical

**Response:** The abstract in the online submission form and manuscript are made identical

4. Please include a copy of Table 4 which you refer to in your text on page 12 and 13

**Response:** A copy of table 4 was included in the original submission; however, due to typing error it was labeled as Table 3. Now a correction is made accordingly.

5. Please include captions for your Supporting Information files at the end of your manuscript, and update any in-text citations to match accordingly

**Response:** Caption for supporting information files have been added at the end of the manuscript and in-text citations are also cross-checked.

6. **Figure 1 copyright issue:**

**Response:** The map of the study area presented in this manuscript is **not copyrighted**. We obtained shape files of ‘Ethio-region’, ‘Districts of Tigray region’, ‘digital elevation model (DEM)’ and ‘road network’ from the data center of the Institute of Climate and Society of Mekelle University and developed the study area map using the applications of ArcGIS. If required we have all the shape files and the ArcGIS file which we can submit for your reference.

III. **Response to Reviewer #1:**

- The language edition was made by a native English speaker (Dr. Matthew, T., associate Professor, College of Veterinary Medicine, Iowa State University), and he has acknowledged in the current version (444-445).
- All the editorial and grammatical issues commented by the reviewers’ on line-by-line bases were addressed as follows:

**Response to comment 1: Line 57** (previous version): the word ‘Taenia’ was misspelt and correction has been made according to the recommendation of the reviewer as ‘taeniasis/cysticercosis’ (**Line 63** in the current version).

**Response to comment 2:** Line 108 (previous version) and **line 123** (current version): The word “that” is replaced with “at” as per the suggestion.

**Response to comment 3:** Line 130 (previous version) and **line 153** (current version): Table 1. Sample size for South-eastern zone is 40 (37 + 3) and 39 was written by typing error. When all the sample sizes are summed considering 40 for S/Eastern zone, it comes 421 which was our sample size.

**Response to comment 4:** Line 301 (previous version) and **line 362-63** (current version): FGD is written in full form.

**Response to comment 5:** Line 303 (previous version) and **line 364** (current version): A full stop is added between Region and Anthrax. The sentence staring with "Anthrax" is also rephrased (**line 364 –65**).

**Response to comment 6:** Line 306 (previous version) and **line 366-68** (current version): …………..public reluctance to vaccinate their animals. The vaccination for anthrax and other major diseases in Ethiopia are coordinated by the government and there is also a significant subsides for vaccines (e.g. the cost of Anthrax vaccine per animal is around 0.01 USD). The reluctance of farmers to vaccinate their animals’ is could be due to lack of awareness on the importance of vaccine.

**Response to comment 7:** Line 314 (previous version) and **line 376** (current version): *M. bovis* is written in full and thank you for your critical observation

**Response to comment 8:** Line 329 (previous version) and **line 391** (current version): the word “and” between Ethiopia and endemic is replaced with “as”.

**Response to comment 9:** Line 330 to 331 (previous version) and **line 392–94** (current version): The sentence beginning “In the present study, brucellosis ………………in other studies [12, 21, 22] is rephrased to become understandable by the reader.

**Response to comment 10:** Line 335 to 338 (previous version) and **line 398-403** (current version): This paragraph is entirely rephrased and improved. Sorry for the confusion.

**Response to comment 11:** Line 345 (previous version) and **line 412** (current version): …”fact that” is deleted from the sentence.

IV. **Response Reviewer #2:**

**Response to comment 1:** In addition to Table 2, barplots for differences in disease type by profession and experience of professionals are added as supporting files (S1 Fig and S2 Fig) and in-text description is also provided.

**Response to comment 2:** line 86 (previous version) and line 92 (current version): 'a' added before 'one health approach'

**Response to comment 3:** lines 108-109 (previous version) and **line 123-25** (current version): use of capital letter for animals was changed into small letters

Biruk Mekonnen (corresponding author)**,** on behalf of all co-authors
