# Supplementary material for: Prioritization of neglected tropical zoonotic diseases: A one health perspective from Tigray region, Northern Ethiopia
Source: PLoS One. 2021 Jul 22;16(7):e0254071. doi: 10.1371/journal.pone.0254071 (PMC8297755; doi:10.1371/journal.pone.0254071)
Supplement: Attachment — Submitted filename: Response to Reviewers.docx [file pone.0254071.s010.docx]

**Ref:** PONE-D-20-30909R1

**Title:** Prioritization of Neglected Tropical Zoonotic Diseases: A One Health Perspective from Tigray Region, Northern Ethiopia

RE: Authors’ responses to reviewers/editors.

Dears,

Thank you for the positive response to our manuscript. We are grateful for the insightful comments of both reviewers and editor. We have addressed the issues raised and proved point by point below and also updated the manuscript with track changes.

1. **Editor’s comment**: Journal requirements on reference lists:

**Answer**: We have reviewed the reference list and found it complete and correct.

2. **Reviewer #1 comments:**

**Comment 1:** Line 42: Delete “s” on challenges

**Revised:** Line 42: *“*… challenge by human health professionals*”*

**Comment 2:** Line 156: Delete “were” between “Who” and “already”

**Revised:** Line 42: *“*… experts who already participated…*”*

**Comment 3:** line 279: Add “and” between production and productivity

**Revised:** Line 279: *“*… low production, and productivity…*”*

**Comment 4:** line 287: Delete the comma after “control”

**Revised:** Line 279: *“*… *treatment, control and prevention programs*…*”*

3. **Reviewer #2 comments:**

**Comment 1:** Table 1 has a minor formatting error in title of third column ni=Ni*n/N)

**Revised:** Line 133: it’s done.

**Comment 2:** the authors should add a brief paragraph on study limitations -e.g. circumstances/available data that limited inferences drawn from the study.

**Revised:** Line 396-405: *“*This study presented some limitations: First, the absence of context specific to one health zoonotic disease prioritization tool could have influenced the prioritization of diseases. In addition, the lack of country-specific data for the majority of the zoonotic diseases has made it difficult to triangulate the results obtained in this study. Second, despite the fact those participants were from diversified disciplines, the number of animal health experts was fewer than human health experts, which may result in bias on professional opinions. In addition, the difference in the background of professionals involved in the study might have influenced the weighing and scoring results of zoonotic diseases. Finally, only 15 accessible districts which had a better road infrastructure and health facility were considered in this study which may not infer the non-accessible districts of the region.*”*
